# A causal association between esophageal cancer and the oral microbiome: a Mendelian randomization study based on an Asian population

**DOI:** 10.3389/fcimb.2024.1420625

**Published:** 2024-09-13

**Authors:** Keke Hu, Ting Huang, Yiming Zhang, Zhifeng Ye, Junhua Guo, Heran Zhou

**Affiliations:** ^1^ Department of Oncology, Hangzhou Traditional Chinese Medicine (TCM) Hospital Affiliated to Zhejiang Chinese Medical University, Hangzhou, Zhejiang, China; ^2^ Clinical Medical Laboratory Center, Jining First People’s Hospital, Shandong First Medical University, Jining, Shandong, China

**Keywords:** oral microbiome, Mendelian randomization, esophageal cancer, genome-wide association studies, Asian population

## Abstract

**Background:**

Previous studies have suggested a crosstalk between the oral microbiome and esophageal cancer (EC), but the exact relationship is unclear. This study aimed to investigate the causal relationship between changes in the oral microbiome and EC by Mendelian randomization (MR).

**Materials and methods:**

In the study, bidirectional MR analyses were conducted using genome-wide association study data from the oral microbiomes from the 4D-SZ cohort and EC data from the BioBank Japan cohort. Multiple sensitivity tests, including Cochrane’s Q statistic, MR-Egger intercept, and MR-PRESSO, were used to assess and validate the relative stability of the resulting data at various levels.

**Results:**

Among the 3,117 samples studied, 73 oral microbiomes were found to be statistically causally associated with EC, 38 of which were considered protective factors. According to species analyses, positive results were concentrated in three phyla: *Firmicutes* (29 species), *Patescibacteria* (18 species), and *Actinobacteria* (9 species). It was also determined that *Parvimonas* micra, *Aggregatibacter*, and *Clostridia* had a negative causal relationship, implying that EC caused a decrease in the counts. Following p-value correction, *periodonticum_C*, *unclassified_mgs_3234*, and *unclassified_mgs_45* were identified as having a strong evidence-grade causal relationship with EC. There was no strong evidence in the results of the inverse MR analyses of EC to the oral microbiome. The sensitivity analysis confirmed the robustness of the findings.

**Conclusion:**

This study discovered a bidirectional causal relationship between the oral microbiome and EC, which may provide new insights into the future use of the microbiome for early screening and probiotic therapy.

## Introduction

According to the global cancer statistics in 2020 conducted by Sung et al, esophageal cancer (EC) ranked 7^th^ in incidence with 3.1% and mortality was 6^th^ at 5.5% among all cancers (Sung et al., 2021). Furthermore, EC is extremely harmful, with an overall 5-year survival rate of only 15.3% (Chitti et al., 2018). For patients who have access to surgery, the 5-year survival rate after esophagectomy is only approximately 40%, indicating a poor prognosis (Markar et al., 2016; Junttila et al., 2023). Histological classification divides EC into two subtypes: esophageal squamous cell carcinoma (ESCC) and esophageal adenocarcinoma (EAC) (Rogers et al., 2022; Ding et al., 2023). The former is typically found in the middle and upper thirds of the esophagus, whereas the latter occurs in the lower third of the esophagus or at the junction with the stomach. EC has been linked to alcohol, smoking, obesity, and diet (Ghosh and Jones, 2022; Ding et al., 2023). However, recent research suggests that the oral microbiome may also play an important role in the incidence of EC. However, an increasing number of studies indicate that the vast oral microbiome plays an important role in this.

The oral microbiome is a large group of microorganisms that live in the oral cavity and has been linked to many human diseases, including dental caries, periodontitis, tooth decay, and peri-esophageal diseases (Wade, 2013; Wu et al., 2023). However, recent studies have found that the oral microbiome is strongly associated with the incidence of many types of cancer. For example, Wang et al. discovered that the risk of ESCC was strongly correlated with the abundance of *Actinomyces* and *Atopobium* (Wang et al., 2019), while another study found that the composition of the oral microbiome could predict the incidence of EC, ESCC, and EAC (Peters et al., 2017). Furthermore, previous studies suggested that the oral microbiome could play a significant role in the pathogenesis of EC. These findings suggest that the oral microbiome may play an important role in the development of oral cancer and other cancers of the digestive tract.

Mendelian randomization (MR) is a method for determining whether there is a causal relationship between an exposure and an outcome (Davies et al., 2018). Natural genetic variation [e.g., single-nucleotide polymorphisms (SNPs)] can be used as an instrumental variable to assess the causal relationship between a biological trait and a disease (Sekula et al., 2016). Although the relationship between the oral microbiome and EC has been studied previously, the causal association between most of the oral microbiome and EC remains unknown. In our study, in the interests of making certain a further causal association between oral microbiome and EC, MR was used to determine the causal relationships between a specific oral microbiome and EC. Furthermore, reverse MR analysis was utilized to determine whether EC could cause any oral changes in microbiome abundance, which could be a critical link between other EC-caused diseases.

## Methods and materials

### Study methods

**Figure 1:**
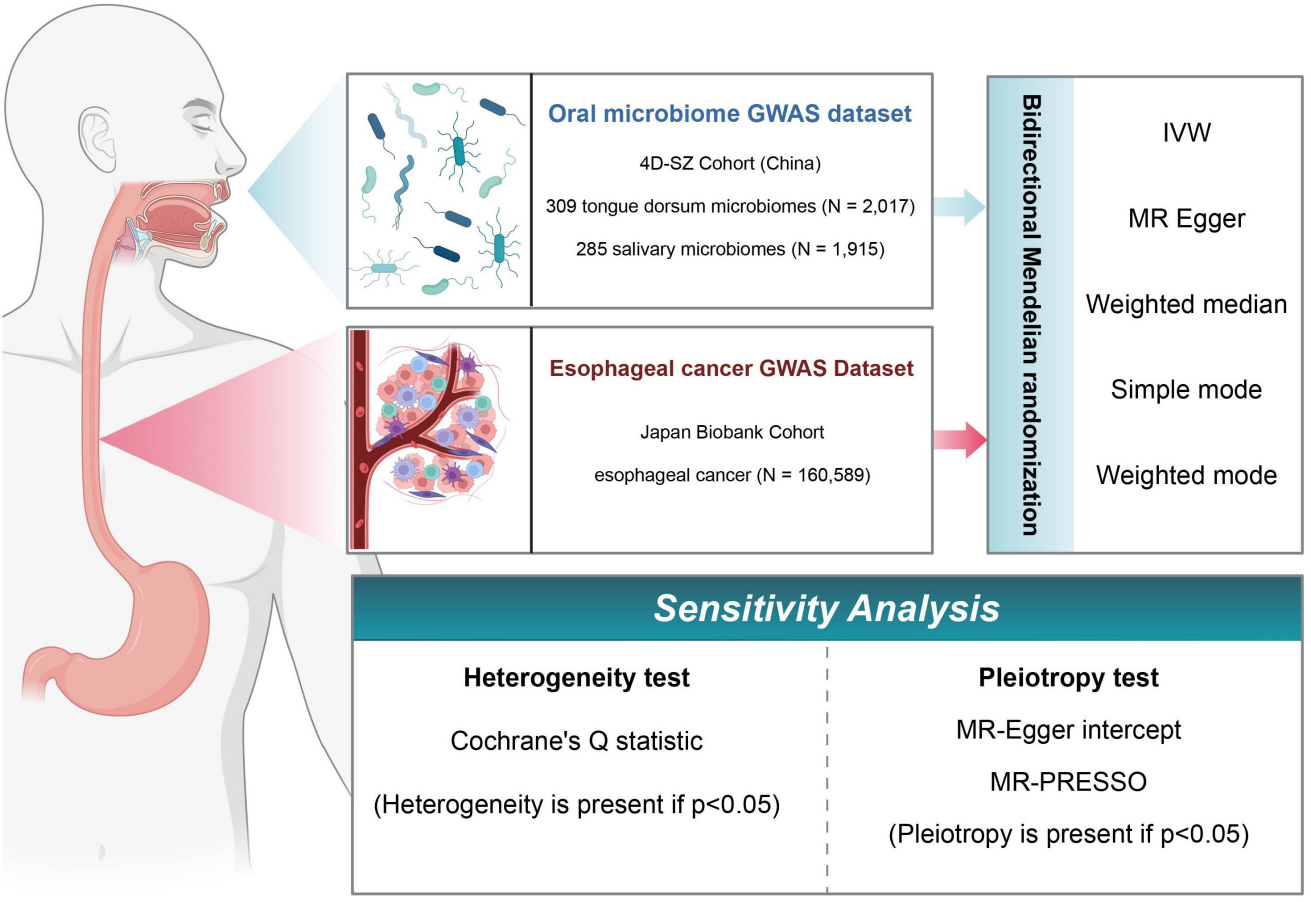
Flowchart of Mendelian randomization (MR) analysis. In this study, the experimental 4D-SZ cohort and the BioBank Japan cohort were subjected to bi-directional MR analysis using a variety of tests, and sensitivity analyses were performed from multiple perspectives.

In this study, we used a two-sample MR framework to extract data from various repositories and investigate the causal relationships between oral microbiome groups in the Shenzhen cohort and EC in the Japanese cohort. Following the identification of positive causality, we used reverse MR to investigate oral microbiome changes in the context of EC. Furthermore, these analyses were subjected to multiple sensitivity analyses to determine the robustness of the findings. A schematic of the study methodology is depicted in **Figure 1**.

### Source of GWAS data for oral microbiome

We used data from the first large-scale genome-wide association study (GWAS), which included 2,017 dorsal tongue samples and 1,915 saliva samples from 2,984 healthy Chinese individuals. There are extensive whole-genome sequencing data available. The 4D-SZ cohort (Multi-Genomics from Shenzhen, China) currently contains high-depth whole-genome sequencing data from 2,984 individuals (average depth of 33×, ranging from 15× to 78×) (Liu et al., 2021). Using 1,583 independent taxa of dorsal tongue microbes (r^2^ < 0.8 from 3,177 taxa using greedy algorithms, materials, and methods) and 10 million human genetic variants (minor allele frequency (MAF) ≥ 0.5%), we found 455 independent associations involving 340 independent loci (distances < 1 Mb, r^2^ < 0.2) and 385 independent taxa reaching genome-wide significance (P < 5 × 10^−8^). The study-wide significance p-value using the more conserved Bonferroni correction was 3.16 × 10^−11^ (= 5 × 10^−8^/1583). There were no additional false positives in the GWAS analyses, and the genome expansion factor λGC ranged from 0.981 to 1.023.

The microbiome composition was determined through comparison with 56,213 macrogenomic assembled genomes. The study examined how host environmental factors to the β-diversity of the oral microbiome (based on genus-level Bray–Curtis differences) was investigated by adjusting for multiple factors including age, gender, body mass index (BMI), diet, lifestyle, substance use, health status issues, and blood measurements. The microbial abundance in both the tongue dorsum and the salivary samples is shown in **Table 1**.

**Table 1:** Relative abundance of oral microbiome phyla in tongue dorsum and saliva samples in original GWAS.

Phyla	Tongue Dorsum	Saliva
*Bacteroidetes*	37.2% ± 11.3%	40.1% ± 10.2%
*Proteobacteria*	30.1% ± 16.5%	30.6% ± 13.1%
*Firmicutes*	20.5% ± 8.2%	17.7% ± 6.7%
*Actinobacteria*	4.3% ± 3.4%	2.6% ± 2.0%
*Fusobacteria*	4.0% ± 1.9%	3.3% ± 1.4%
*Patescibacteria*	2.5% ± 1.6%	3.1% ± 1.6%
*Campylobacterota*	1.1% ± 0.9%	1.3% ± 0.8%

The entire community had 99.7% coverage in the tongue dorsum samples and 98.7% in the saliva samples. The 16s rRNA gene amplicon sequencing of the HMP results confirmed that saliva samples had greater alpha diversity than tongue dorsum samples. The representative mean Shannon index was 6.476 versus 6.228 Wilcoxon rank sum test P < 2.2 × 10^−16^.

### Sources of GWAS data for esophageal cancer

GWAS data for EC were obtained from 220 deep phenotype genome-wide association studies (disease, biomarker, and drug use) conducted at BioBank Japan (n = 179,000) (Sakaue et al., 2021). The map revealed the pleiotropic landscape represented by major histocompatibility complex loci and the sites where HLA fine targeting was performed in a high-quality genetic linkage group dataset from an East Asian population. The large-scale non-European population genetic association map was chosen for this study. A total of 160,589 sample sizes were collected for EC data, with 12,455,381 SNPs included. After de-chaining the imbalance, we adjusted for gender, year of birth, genotyping batch, and the first four principal components. The annotated version was created with HG19/GRCh37.

### Statistical analysis

We primarily used inverse variance weighting (IVW) with multiplicative random effects to combine SNP-specific Wald estimates (outcome SNP divided by exposure SNP) to generate odds ratios (ORs) or beta coefficients (mean differences) for 95% confidence intervals (CIs), Cochrane’s Q statistic, and p-values for heterogeneity (Bowden et al., 2017). The independent variables were selected using the “cluster data” function of the MR-Base r package (r^2^ < 0.001). Non-allelic or non-allelic variants, as well as variants with missing rs numbers, were excluded. For sensitivity analyses to evaluate horizontal pleiotropy, we used three complementary methods with distinct assumptions about validity estimates: 1) weighted median, which extracted data for valid SNPs above 50%; 2) the MR-Egger method, which allowed for all SNPs to be invalid under the assumption of InSIDE (Instrumental Strength Independence of Direct Effects), where a p < 0.05 intercept indicates the presence of pleiotropy; 3) Mendelian randomization of polytropic residuals and outliers (MR-PRESSO) which identifies potential polytropic outliers, and the data are cleaned and estimated after these anomalous SNPs are removed (Hemani et al., 2018).

In the Shenzhen Oral Microbiome Project in China, we used MR analysis of each microbiome after de-chaining imbalance as a general screening method to identify causal relationships. We then performed MR analysis on the cleaned data from 3,117 oral microbiomes. We used a threshold of p < 5 × 10^−8^ for each SNP to extract meaningful study ranges (p < 3.16 × 10^−11^) among the five identified genetic loci associated with the oral microbiome. We primarily used the IVW method, but we also used the MR-Egger, weighted median, weighted model, and simple median methods (Bowden et al., 2015; Burgess et al., 2015; Bowden et al., 2016; Hartwig et al., 2017; Sanderson et al., 2019). To ascertain the statistical validity of the MR analyses, the ORs and corresponding confidence intervals for the correlations between individual oral microorganisms and the study results were evaluated using the Wald ratio and the delta method, a generalized method for deriving variance MR correlations by scaling, i.e., for every standard deviation increase in the risk of genetically predicted oral microbial trends, the MR correlation increased by one standard deviation. The study conducted MR analysis using TwoSample MR and the Mendelian randomization package in R software (4.3.2).

## Results

### MR analysis of oral microbiome to esophageal cancer

**Figure 2:**
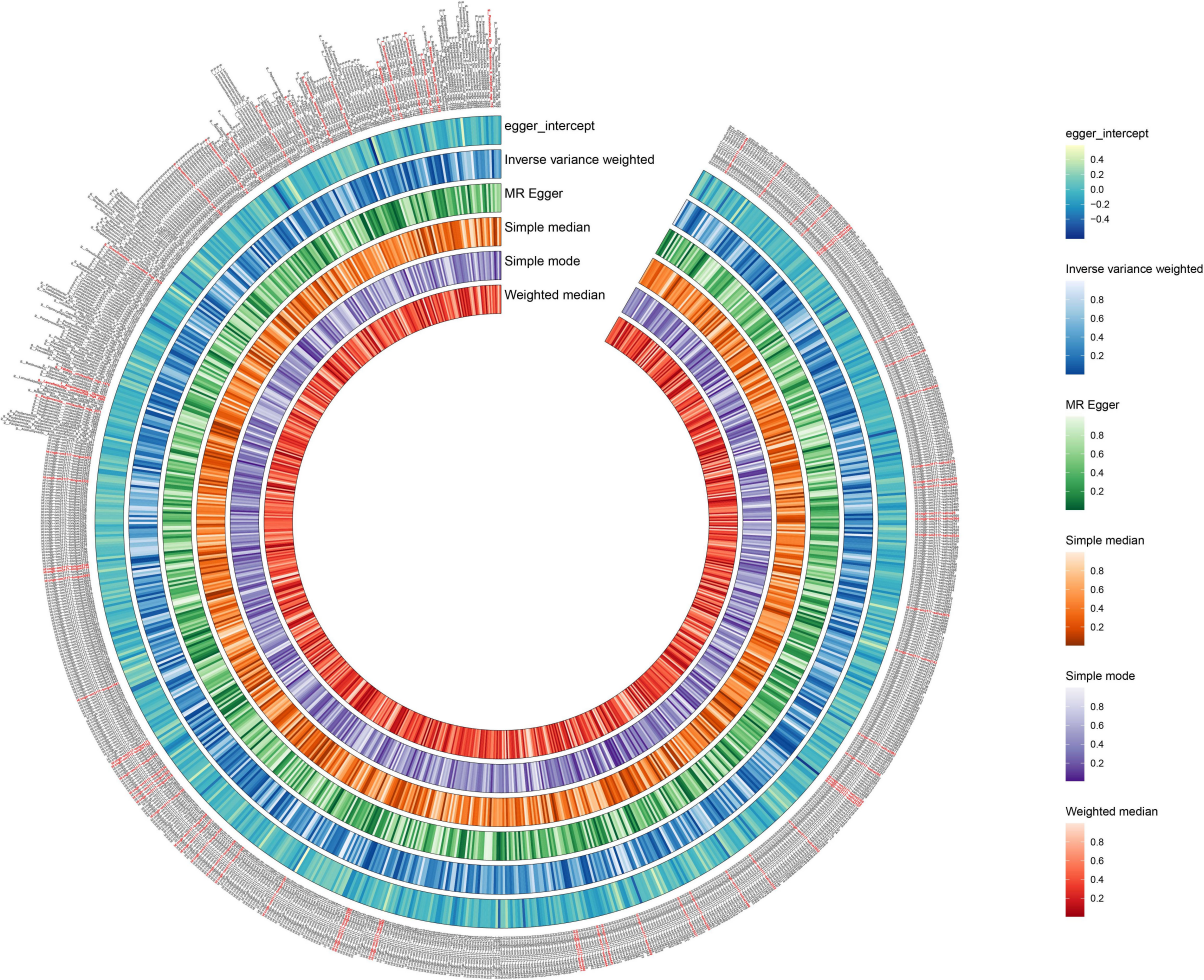
Positive results of screening oral microbiomes to esophageal cancer (EC). The circular plot shows the relationship between oral microbiome species and EC using five Mendelian randomization methods: Inverse Variance Weighted, MR Egger, Simple Median, Simple Mode, and Weighted Median. A p-value less than 0.05 was considered statistically significant and these results are highlighted in red.

**Figure 3:**
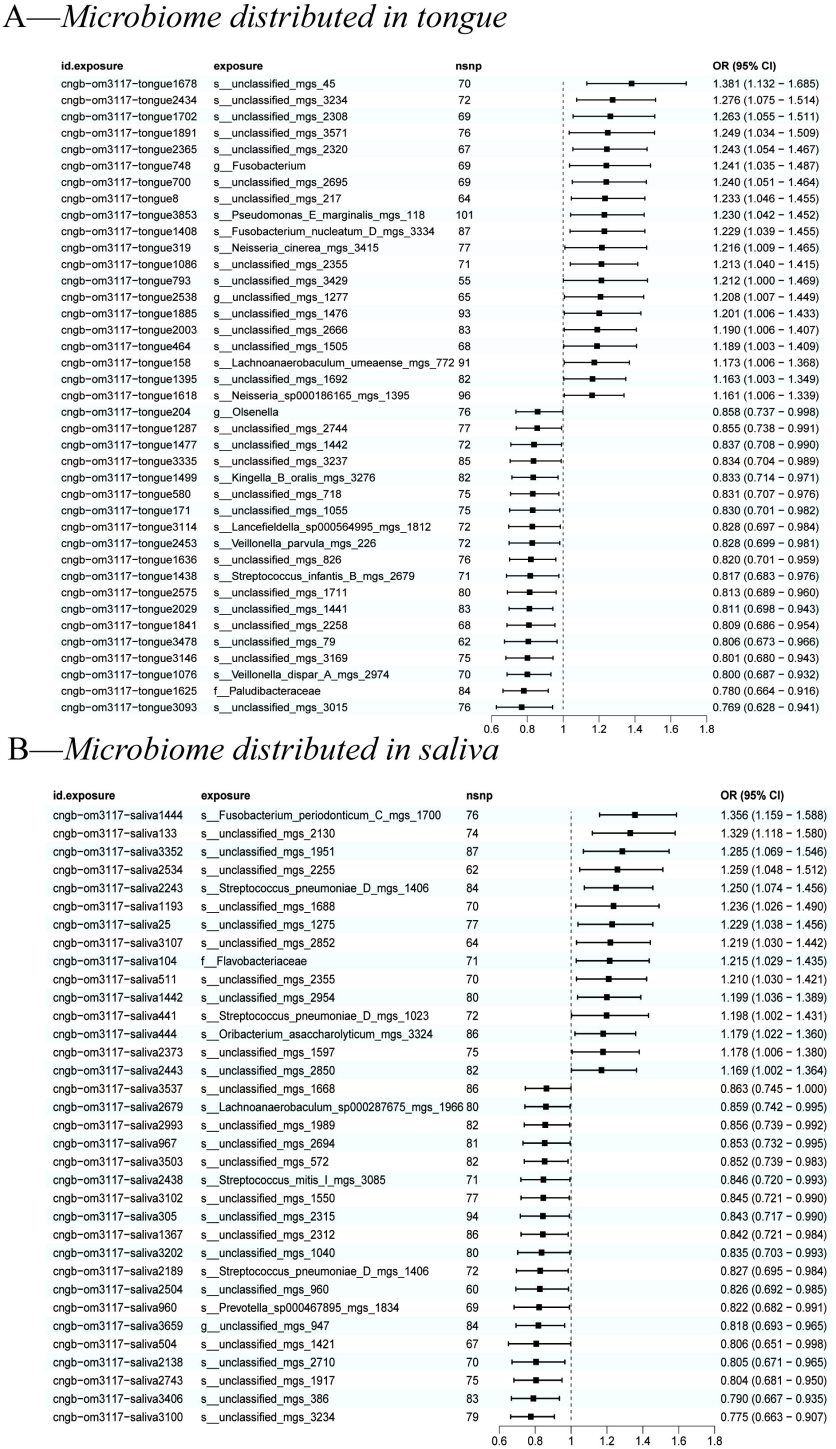
A forest plot of positive results of oral microbiomes to esophageal cancer (EC). **(A)** Positive results of tongue-sourced oral microbiomes to EC. **(B)** Positive results of saliva-derived oral microbiomes to EC. The results were mainly evaluated by the IVW method and a p-value less than 0.05 was considered statistically significant.

We used the IVW method as the primary indicator of the MR analysis results and identified 73 positive oral microbiome exposures with a causal association with EC based on their p-values (**Figure 2**). There were 39 oral microbiomes distributed in the tongue samples, and 34 oral microbiomes distributed in the saliva samples (**Figure 3**).

All positive associations between the oral microbiome and esophageal cancer are listed in **Table 2**, ordered by p-value from significant to highly significant. Among the oral microbiomes found in the tongue dorsum samples, 19 species were protective factors against esophageal cancer, while 20 species were risk factors. Among the oral microbiomes found in the saliva samples, 19 species were protective factors against esophageal cancer, while 15 species were risk factors.

**Table 2:** Association of oral microbiome species with esophageal cancer: positive results ranked by P-value.

Species	OR (95% CI)	P-value
Protective Association of Tongue-Derived Oral Microbiome Species with Esophageal Cancer		
*Olsenella*	0.858 (0.737 - 0.998)	4.73E-02
*unclassified_2744 in Streptococcus*	0.855 (0.738 - 0.991)	3.79E-02
*unclassified_1442 in Prevotella*	0.837 (0.708 - 0.990)	3.76E-02
*unclassified_3237 in TM7x*	0.834 (0.704 - 0.989)	3.65E-02
*sp000564995*	0.828 (0.697 - 0.984)	3.24E-02
*unclassified_1055 in Streptococcus*	0.830 (0.701 - 0.982)	2.96E-02
*parvula*	0.828 (0.699 - 0.981)	2.91E-02
*infantis_B*	0.817 (0.683 - 0.976)	2.61E-02
*unclassified_718 in Streptococcus*	0.831 (0.707 - 0.976)	2.42E-02
*oralis*	0.833 (0.714 - 0.971)	1.98E-02
*unclassified_79 in Treponema_D*	0.806 (0.673 - 0.966)	1.94E-02
*unclassified_1711 in UBA2866*	0.813 (0.689 - 0.960)	1.47E-02
*unclassified_826 in Saccharimonadaceae*	0.820 (0.701 - 0.959)	1.30E-02
*unclassified_2258 in Streptococcus*	0.809 (0.686 - 0.954)	1.18E-02
*unclassified_mgs_3015 in TM7x*	0.769 (0.628 - 0.941)	1.08E-02
*unclassified_3169 in TM7x*	0.801 (0.680 - 0.943)	7.73E-03
*unclassified_1441 in Gemella*	0.811 (0.698 - 0.943)	6.34E-03
*dispar_A*	0.800 (0.687 - 0.932)	4.11E-03
*Paludibacteraceae*	0.780 (0.664 - 0.916)	2.51E-03
Risk Association of Tongue-Derived Oral Microbiome Species with Esophageal Cancer		
*unclassified_3429 in Granulicatella*	1.212 (1.000 - 1.469)	4.95E-02
*unclassified_1505 in Oribacterium*	1.189 (1.003 - 1.409)	4.63E-02
*unclassified_1692 in Catonella*	1.163 (1.003 - 1.349)	4.49E-02
*unclassified_1476 in F0040*	1.201 (1.006 - 1.433)	4.26E-02
*unclassified_2666 in Lancefieldella*	1.190 (1.006 - 1.407)	4.24E-02
*unclassified_1277 in Saccharimonadaceae*	1.208 (1.007 - 1.449)	4.23E-02
*umeaense*	1.173 (1.006 - 1.368)	4.21E-02
*sp000186165*	1.161 (1.006 - 1.339)	4.08E-02
*cinerea*	1.216 (1.009 - 1.465)	3.97E-02
*unclassified_3571 in Pauljensenia*	1.249 (1.034 - 1.509)	2.13E-02
*Fusobacterium*	1.241 (1.035 - 1.487)	1.95E-02
*nucleatum_D*	1.229 (1.039 - 1.455)	1.64E-02
*E_marginalis*	1.230 (1.042 - 1.452)	1.46E-02
*unclassified_2355 in Fusobacterium*	1.213 (1.040 - 1.415)	1.42E-02
*unclassified_217 in Eikenella*	1.233 (1.046 - 1.455)	1.28E-02
*unclassified_2695 in Saccharimonadaceae*	1.240 (1.051 - 1.464)	1.10E-02
*unclassified_2308 in Pauljensenia*	1.263 (1.055 - 1.511)	1.08E-02
*unclassified_2320 in Streptococcus*	1.243 (1.054 - 1.467)	9.88E-03
*unclassified_3234 in CAG-793*	1.276 (1.075 - 1.514)	5.25E-03
*unclassified_45 in UBA6648*	1.381 (1.132 - 1.685)	1.50E-03
Protective Association of Saliva-Derived Oral Microbiome Species with Esophageal Cancer		
*unclassified_1668 in TM7x*	0.863 (0.745 - 1.000)	4.93E-02
*unclassified_1421 in Granulicatella*	0.806 (0.651 - 0.998)	4.80E-02
*unclassified_2694 in Fusobacterium*	0.853 (0.732 - 0.995)	4.24E-02
*sp000287675*	0.859 (0.742 - 0.995)	4.23E-02
*unclassified_1040 in Stomatobaculum*	0.835 (0.703 - 0.993)	4.16E-02
*mitis_I*	0.846 (0.720 - 0.993)	4.13E-02
*sp000467895*	0.822 (0.682 - 0.991)	3.95E-02
*unclassified_1989 in Pauljensenia*	0.856 (0.739 - 0.992)	3.88E-02
*unclassified_2315 in Fusobacterium*	0.843 (0.717 - 0.990)	3.78E-02
*unclassified_1550 in Lancefieldella*	0.845 (0.721 - 0.990)	3.72E-02
*unclassified_960 in Campylobacter_A*	0.826 (0.692 - 0.985)	3.36E-02
*pneumoniae_D*	0.827 (0.695 - 0.984)	3.24E-02
*unclassified_2312 in Fusobacterium*	0.842 (0.721 - 0.984)	3.05E-02
*unclassified_572 in TM7x*	0.852 (0.739 - 0.983)	2.81E-02
*unclassified_2710 in Streptococcus*	0.805 (0.671 - 0.965)	1.93E-02
*unclassified_947 in Saccharimonadaceae*	0.818 (0.693 - 0.965)	1.70E-02
*unclassified_1917 in Campylobacter_A*	0.804 (0.681 - 0.950)	1.02E-02
*unclassified_386 in TM7x*	0.790 (0.667 - 0.935)	6.18E-03
*unclassified_3234 in CAG-793*	0.775 (0.663 - 0.907)	1.43E-03
Risk Association of Saliva-Derived Oral Microbiome Species with Esophageal Cancer		
*unclassified_2850 in Streptococcus*	1.169 (1.002 - 1.364)	4.73E-02
*pneumoniae_D*	1.198 (1.002 - 1.431)	4.70E-02
*unclassified_1597 in Campylobacter_A*	1.178 (1.006 - 1.380)	4.23E-02
*unclassified_1688 in CAG-793*	1.236 (1.026 - 1.490)	2.58E-02
*asaccharolyticum*	1.179 (1.022 - 1.360)	2.43E-02
*Flavobacteriaceae*	1.215 (1.029 - 1.435)	2.18E-02
*unclassified_2852 in Pauljensenia*	1.219 (1.030 - 1.442)	2.10E-02
*unclassified_2355 in Fusobacterium*	1.210 (1.030 - 1.421)	2.03E-02
*unclassified_1275 in Neisseria*	1.229 (1.038 - 1.456)	1.68E-02
*unclassified_2954 in Pauljensenia*	1.199 (1.036 - 1.389)	1.49E-02
*unclassified_2255 in Catonella*	1.259 (1.048 - 1.512)	1.40E-02
*unclassified_1951 in TM7x*	1.285 (1.069 - 1.546)	7.73E-03
*pneumoniae_D_1406*	1.250 (1.074 - 1.456)	4.02E-03
*unclassified_2130 in CAG-793*	1.329 (1.118 - 1.580)	1.25E-03
*periodonticum_C*	1.356 (1.159 - 1.588)	1.49E-04

### Sensitivity analysis of oral microbiome to esophageal cancer

We used MR-PRESSO, MR-Egger intercept, and Cochran’s Q test to assess the pleiotropy and heterogeneity of the MR analysis results to ensure that non-robust results did not influence the conclusions. MR-PRESSO identified the presence of *unclassified_mgs_3015* and *infantis_B* horizontal pleiotropy; MR-Egger intercept detected pleiotropy only in *unclassified_mgs_2852* out of the 73 positive results; and Cochran’s Q test revealed heterogeneity in *unclassified_mgs_3015* and *unclassified_mgs_2130*. We used IVW’s random effects model for *infantis_B*, *unclassified_mgs_3015*, and *unclassified_mgs_2130* to avoid bias in the above data, and the results show that the above results remain significant when using the random effects model (**Supplementary Tables 1**, **2**).

### MR analysis of esophageal cancer to the oral microbiome

To rule out reverse causality from the oral microbiome to EC and investigate whether a causal association exists between EC and the oral microbiome, we conducted reverse causality with EC as the exposure and the oral microbiome as the outcome (**Table 3**). The findings indicated that no reverse causality was previously found in 73 positive oral microbiome exposures, in contrast to the three oral microbiomes that were identified to have reduced abundance in EC: *Parvimonas micra* (p = 0.048, OR = 0.96, 95% CI = 0.91–1.00), *Aggregatibacter* (p = 0.042, OR = 0.95, 95% CI = 0.91–1.00), and *Clostridia* (p = 0.042, OR = 0.96, 95% CI = 0.91–1.00).

**Table 3:** Reverse MR of the relationship between the oral microbiome and esophageal cancer.

Bacterial taxa (Outcome)	MR method	No. of SNP	F-Power	OR	95% CI	P-value
*Parvimonas micra*	IVW	3	32	0.96	0.91—1.00	0.048*
	MR Egger	3		0.94	0.83—1.07	0.541
	Weighted median	3		0.96	0.90—1.02	0.080
	Weighted mode	3		0.95	0.91—1.00	0.280
	Simple mode	3		0.95	0.89—1.02	0.280
*Aggregatibacter*	IVW	3	31	0.95	0.91—1.00	0.042*
	MR-Egger	3		0.94	0.82—1.07	0.514
	Weighted median	3		0.95	0.89—1.02	0.145
	Weighted mode	3		0.95	0.90—1.01	0.078
	Simple mode	3		0.95	0.89—1.01	0.272
*Clostridia*	IVW	3	32	0.96	0.91—1.00	0.042*
	MR-Egger	3		0.95	0.83—1.08	0.594
	Weighted median	3		0.96	0.89—1.02	0.178
	Weighted mode	3		0.96	0.91—1.00	0.067
	Simple mode	3		0.95	0.89—1.02	0.309

*Bolded font indicates a p-value less than 0.05.

### Sensitivity analysis of esophageal cancer to oral microbiome

**Figure 4:**
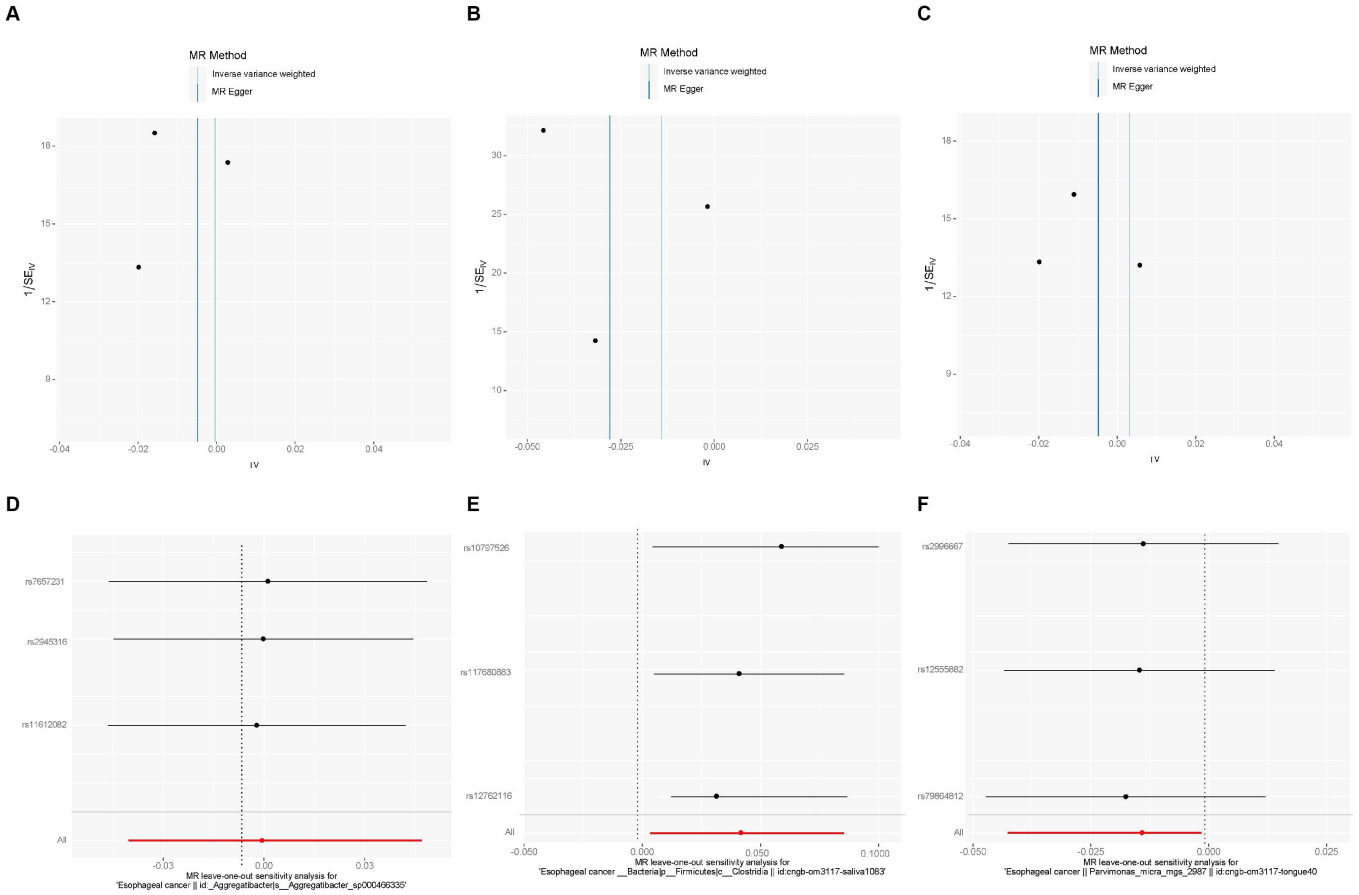
A funnel plot with the leave-one-out method for MR analysis of EC to oral microbiomes. **(A-C)** Funnel plot. **(D-F)** Leave-one-out method. Funnel plots are commonly used to detect bias. The x-axis represents the degree of variation, while the y-axis typically represents either sample size or total effect size. The goal is to observe whether the SNPs are symmetrically distributed on both sides of the IVW line (in light blue) and MR Egger line (in deep blue); a symmetrical distribution indicates no bias. The leave-one-out analysis involves sequentially removing each SNP and performing MR analysis. The figure shows that the funnel plot is symmetrical on both sides of the SNPs, and the leave-one-out method produces a smooth result curve after each sample is removed, indicating stable evidence.

We also used MR-PRESSO, MR-Egger intercept, and Cochran’s Q test for sensitivity analyses of the results, which revealed that the p-values were greater than 0,05, indicating that none of the three causalities had horizontal pleiotropy, pleiotropy, and heterogeneity (**Table 4**). We used the funnel plot with the leave-one-out method as a secondary reference to assess the robustness of the results. **Figure 4** shows that the funnel plot is symmetrical on both sides of the SNP, and the leave-one-out method produced a smooth result curve after removing each sample, indicating that the evidence is stable (**Figure 4**).

**Table 4:** Sensitivity analysis of esophageal cancer to oral microbiome.

Bacterial taxa (Outcome)	MR-PRESSO global test*		MR-Egger intercept p-Egger			Cochran’s Q test	
	MR-PRESSO RSSobs	P-value	Egger-intercept	Standard Error	P-value	IVW (P)	MR-Egger p
*Parvimonas micra*	23.21	0.313	0.028	0.065	0.595	0.643	0.035
*Aggregatibacter*	27.13	0.427	0.025	0.066	0.514	0.896	0.475
*Clostridia*	25.33	0.248	0.023	0.065	0.541	0.387	0.233

*Data from the MR-PRESSO global test, MR-Egger intercept, and Cochran’s Q test sensitivity and heterogeneity tests for reverse MR.

### P-value correction

We used the false discovery rate (FDR) correction for the Benjamini–Hochberg (BH) method to correct the p-values for the 73 positive results that previously identified oral microbiome exposure to EC, as well as the 3 positive results for EC exposure to oral microbiomes. We classified a result as having a strong level of evidence if the p-value remained significant after FDR correction, and as having a weak level of evidence if the p-value had vanished after FDR correction.

**Figure 5:**
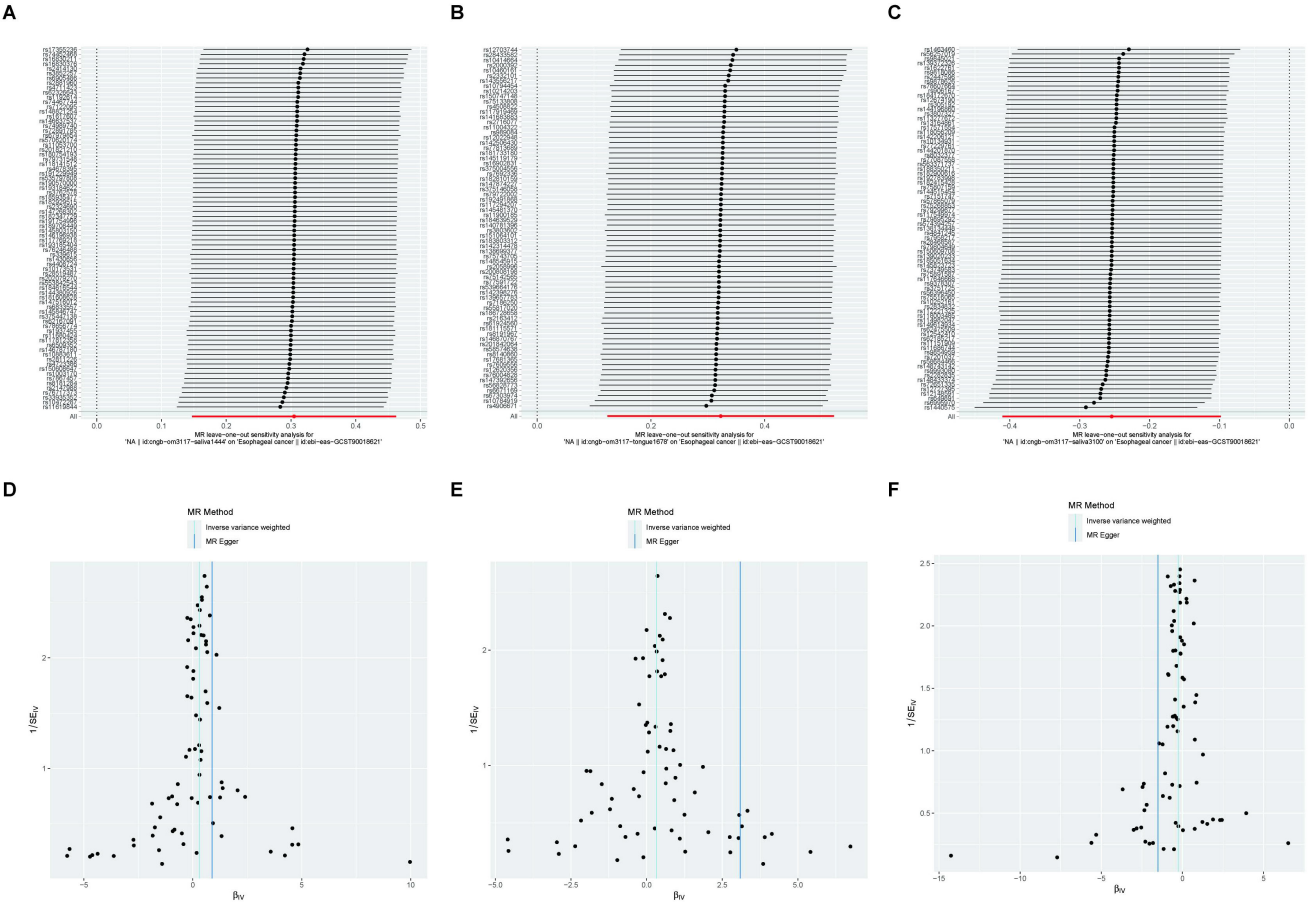
A funnel plot of the MR analysis of oral microbiomes to EC with the leave-one-out method. **(A-C)** Funnel plot. **(D-F)** Leave-one-out method. Funnel plots are commonly used to assess bias. The x-axis represents the degree of variation, while the y-axis typically represents either sample size or total effect size. The goal is to observe whether the SNPs are symmetrically distributed on both sides of the IVW line (in light blue) and the MR Egger line (in deep blue); a symmetrical distribution indicates no bias. The leave-one-out analysis involves sequentially removing each SNP and performing MR analysis. The symmetry observed in the funnel plot using the leave-one-out method results in a smooth curve after each sample is removed, further demonstrating the stability of the evidence.

The results revealed that after adjusting for FDR, there were still three oral microbiomes with significant causal relationships with EC: *periodonticum_C* (P_adj_ = 0.013), *unclassified_mgs_3234* in *CAG-793* (P_adj_ = 0.031), and *unclassified_mgs_45* in *UBA6648* (P_adj_ = 0.043). *Periodonticum_C* and *unclassified_mgs_45* were risk factors, while *unclassified_mgs_3234* was protective. *Periodonticum_C* and *unclassified_mgs_3234* originated in the saliva samples, while *unclassified_mgs_45* originated in the tongue samples. The sensitivity analysis revealed that none of the three oral microbiomes mentioned above exhibited pleiotropy or heterogeneity (**Table 5**). Furthermore, the symmetry of the SNP sides of the funnel plot using the leave-one-out method yielded a smooth curve of results after removing each sample, again demonstrating the stability of the evidence (**Figure 5**).

**Table 5:** Sensitivity analysis of oral microbiome to esophageal cancer.

Bacterial taxa (exposure)	MR-Egger intercept p-Egger			Cochran’s Q test		MR-PRESSO global test	
	egger_intercept	SE	Pval	MR-Egger (p)	IVW(P)	MR-PRESSO RSSobs	Pval
*unclassified_mgs_3234*	0.120	0.152	0.434	0.561	0.573	76.789	0.598
*periodonticum_C*	−0.057	0.153	0.709	0.950	0.957	56.462	0.966
*unclassified_mgs_45*	−0.265	0.194	0.176	0.849	0.826	58.977	0.857

The p-values of the three positive results for EC exposure to the oral microbiome were no longer significant following correction, and all were positive results with a low level of evidence.

## Discussion

The human oral microbiome is the second most diverse in terms of species after the gut microbiome (Baker et al., 2024). Most researchers recognize the interaction of the oral microbiome with the host’s immune system, as well as its impact on both systems’ health (Gao et al., 2018; Fan et al., 2023). Furthermore, an increasing number of studies suggest a close link between the oral microbiome and the development of various cancers (Feng et al., 2023). However, due to its complex species composition and diverse individual variations (Tierney et al., 2019), the mechanisms of interaction between the oral microbiome and cancer development have yet to be thoroughly explained and illustrated. Asia has a high incidence of EC, particularly ESCC, and there is even an “Asian esophageal cancer belt” stretching from Central to East Asia (Zhang et al., 2012; Zhu et al., 2023). Therefore, the current study investigated the causal relationship between the oral microbiome and EC development in an Asian population using bidirectional MR. There was some meaningful statistical evidence suggesting a causal role for the 73 oral microbiomes in developing EC and a causal effect on the three oral microbiomes after EC development, while sensitivity analyses revealed no pleiotropy or heterogeneity in any of the results. We then used the FDR correction for the BH method to adjust the p-values for the bidirectional results. The results revealed that after correcting for FDR, three oral microbiomes had significant causal relationships with EC: *periodonticum_C*, *unclassified_mgs_3234* in *CAG-793*, and *unclassified_mgs_45* in *UBA6648*. However, after correction, the p-values for the three positive results for EC exposure to the oral microbiome were no longer significant, indicating that all of the positive results were supported by weak evidence.

When meticulously analyzing the distribution of the results, it is first observed that the distribution was relatively homogeneous in the tongue samples (39 species) and the saliva samples (34 species). Second, at the phylum level, positive results were found in three phyla: *Firmicutes* (29 species), *Patescibacteria* (18 species), and *Actinobacteria* (9 species). A 16s sequencing study of the oral microbiome by Jiang et al. also found that *Firmicutes*, *Actinobacteria*, and *Patescibacteria* were superior in ESCC patient samples (Jiang et al., 2023), which supports our statistical findings. Overall, the orders, phyla, family, and genera with the most widespread oral microbiomes causally associated with EC are *Bacilli*, *Lactobacillales*, *Streptococcaceae*, and *Streptococcus*. It is worth noting that, according to the MR analysis, 38 of the 73 positive oral microbiomes were thought to play a protective role, accounting for approximately 52.1% of the total, with 19 being salivary and 19 being tongue-derived. Of the three FDR-tested supportive results, *periodonticum_C* and *unclassified_mgs_3234* from the saliva samples were considered as protective and risk factors, respectively, while *unclassified_mgs_45* from tongue sources was classified as protective. Interestingly, eight of the 12 species in the genus *Streptococcus* are thought to be resistant to EC, including *Streptococcus pneumoniae*. This appears to contradict the widely held belief that *Streptococcus* is harmful to humans (Papadimitriou, 2018), implying a more complex network of roles in the interaction of the oral microbiome and disease. TM7X is another genus of oral microbiome that is thought to play a primarily protective role in the family *Saccharimonadaceae* (Baker et al., 2017). Our study found that seven of the eight positive TM7X results were considered protective. Zhang et al. discovered that TM7X genus levels were significantly elevated in non-tumor tissues from patients with ESCC at stage T4 (Zhang et al., 2022). These findings suggest that TM7X may have a previously unexplored protective mechanism in digestive system cancers, with significant research value. For example, could probiotic drugs based on the TM7X genus improve prognosis in EC or assess disease progression through changes in expression?

Furthermore, when focusing on changes in the oral microbiome of patients who had developed EC, *Parvimonas micra*, *Aggregatibacter*, and *Clostridia* showed a negative causal relationship, implying that EC causes a decrease in the abundance of these three bacteria. *Clostridia* is a genus of gram-positive bacilli, anaerobic bacteria that are involved in food fermentation and the production of beneficial metabolites (Patakova et al., 2022). There are studies indicating that cultures of *Clostridium butyricum* can inhibit colorectal cancer in mice (Pu et al., 2023), and the use of *Clostridium butyricum* therapy can enhance the efficacy of immune checkpoint blockade therapy in lung cancer (Tomita et al., 2022). These findings support our finding of the protective role of *Clostridia* in EC. In contrast, available studies on *Parvimonas micra* and *Aggregatibacter* appear to indicate that their increased abundance is a risk factor for certain tumors. For example, *Parvimonas micra* is thought to be associated with reduced survival in colorectal cancer (CRC) patients and may promote CRC progression by activating the Ras/ERK/c-Fos pathway (Zhao et al., 2022; Chang et al., 2023). *Aggregatibacter* is a causative organism of oral diseases such as gingivitis and periodontitis, which frequently causes oral and systemic diseases, chronic inflammation in the host, and may cause cancer (Coussens and Werb, 2002; Zhang et al., 2019). Given the differences observed between our study and previous research regarding the relationship between *Parvimonas micra* and *Aggregatibacter* with EC which may be attributed to methodological variations, further validation is essential.

In the current scenario of rising antimicrobial resistance, probiotics have garnered significant attention due to their ability to modulate the gut and oral microbiome, promote the growth of beneficial bacteria, and inhibit pathogenic microorganisms, thereby playing a crucial role in the prevention and treatment of various diseases (Sanders et al., 2019). For patients with cancer, probiotics can enhance anti-tumor immune responses by influencing gut barrier function and regulating immune cell activity, serving as a vital alternative to antibiotics. Additionally, probiotics possess anti-inflammatory properties as they can suppress chronic inflammation, a key factor in the development of esophageal cancer, by reducing the production of pro-inflammatory cytokines such as IL-6 and TNF-a, thus slowing or preventing cancer progression. Furthermore, probiotics can help reconstruct the gut and oral microbiome, often disrupted by treatments such as radiotherapy and chemotherapy, restoring the balance of beneficial bacteria, reducing harmful microorganisms, and improving overall patient health (Mego et al., 2013; Co et al., 2023). Future research should focus on systematically screening and identifying the most effective probiotic strains, especially in high-incidence regions of EC in Asia, conducting large-scale randomized controlled trials to determine the specific effects and optimal usage of probiotics, and investigating their combined use with conventional therapies to enhance therapeutic effects and reduce side effects.

In conclusion, bidirectional MR revealed significant associations between the 73 oral microbiomes and oral cancer. After p-value correction, three types of strong evidence-grade relationships for EC were identified, but no strong evidence was found in the results of the inverse MR analyses of EC to the oral microbiome. However, the research methodology utilized in this paper has some flaws. The first step is in the selection of the samples. Because of the high incidence of EC in Asia, the subjects chosen for this study were Asian populations, and by using two population cohorts that were ethnically consistent and not genetically related to each other, biasing the results was avoided, thereby strengthening the paper’s conclusions. However, sample selection suffered from an over-concentration of sources, with only East Asian regions such as China and Japan represented. More diverse patient data from other parts of Asia are required to supplement with Asia-wide clinical information. Meanwhile, the number of GWAS samples included in this study was limited, with only 2,017 dorsal tongue samples and 1,915 saliva samples from 2,984 healthy Chinese individuals. In the future, the data source should be expanded to include multinational centers. Furthermore, it should be noted that the causality identified in MR studies is only statistical, and the conclusions reached using this method must be combined with the results of actual experimental validation. Finally, we comprehensively examined the potential causal relationship between the oral microbiome and EC. As the first MR study of the oral microbiome and EC in the known range, this paper offers an intriguing new direction and possibility for the study of interactions between oral microbes and cancer.

## Data Availability

The original contributions presented in the study are included in the article/supplementary material. Further inquiries can be directed to the corresponding author.
